# Effects of Tungsten Addition on the Microstructure and Properties of FeCoCrNiAl High-Entropy Alloy Coatings Fabricated via Laser Cladding

**DOI:** 10.3390/ma17143592

**Published:** 2024-07-20

**Authors:** Shibang Ma, Congzheng Zhang, Liang Li, Haodong Chen, Yinhai Yang

**Affiliations:** School of Intelligent Manufacturing and Electrical Engineering, Nanyang Normal University, Nanyang 473061, China; zcz1010@126.com (C.Z.); liang0320@126.com (L.L.); chd1230@126.com (H.C.); nynuyyh@163.com (Y.Y.)

**Keywords:** laser cladding, high-entropy alloy, FeCoCrNiAl, W, microstructure, wear resistance, corrosion resistance

## Abstract

This study examines the effects of different addition levels of tungsten (W) content on the microstructure, corrosion resistance, wear resistance, microhardness, and phase composition of coatings made from FeCoCrNiAl high-entropy alloy (HEA) using the laser cladding technique. Using a preset powder method, FeCoCrNiAlW*_x_* (where *x* represents the molar fraction of W, *x* = 0.0, 0.2, 0.4, 0.6, 0.8) HEA coatings were cladded onto the surface of 45 steel. The different cladding materials were tested for dry friction by using a reciprocating friction and wear testing machine. Subsequently, the detailed analysis of the microstructure, phase composition, corrosion resistance, wear traces, and hardness characteristics were carried out using a scanning electron microscope (SEM), X-ray diffractometer (XRD), electrochemical workstation, and microhardness tester. The results reveal that as the W content increases, the macro-morphology of the FeCoCrNiAlW*_x_* HEA cladding coating deteriorates; the microstructure of the FeCoCrNiAlW*_x_* HEA cladding coating, composed of μ phase and face-centered cubic solid solution, undergoes an evolution process from dendritic crystals to cellular crystals. Notably, with the increase in W content, the average microhardness of the cladding coating shows a significant upward trend, with FeCoCrNiAlW_0.8_ reaching an average hardness of 756.83 HV_0.2_, which is 2.97 times higher than the 45 steel substrate. At the same time, the friction coefficient of the cladding coating gradually decreases, indicating enhanced wear resistance. Specifically, the friction coefficients of FeCoCrNiAlW_0.6_ and FeCoCrNiAlW_0.8_ are similar, approximately 0.527. The friction and wear mechanisms are mainly adhesive and abrasive wear. In a 3.5 wt.% NaCl solution, the increase in W content results in a positive shift in the corrosion potential of the cladding coating. The FeCoCrNiAlW_0.8_ exhibits a corrosion potential approximately 403 mV higher than that of FeCoCrNiAl. The corrosion current density significantly decreases from 5.43 × 10^−6^ A/cm^2^ to 5.26 × 10^−9^ A/cm^2^, which suggests a significant enhancement in the corrosion resistance of the cladding coating.

## 1. Introduction

The alloy 45 steel, known for its excellent comprehensive mechanical properties, high tensile strength, and relatively high deformation resistance, is extensively utilized in various fields such as machine tools, automobiles, marine equipment, and agricultural machinery [1]. However, the failure of machine components primarily stems from wear and corrosion [2], limiting the widespread performance of 45 steel under specific working conditions due to its relatively poor surface wear resistance and corrosion resistance. Replacing 45 steel with materials possessing higher wear and corrosion resistance would incur significant costs. An effective solution to this problem is using surface cladding technology to coat the steel surface with wear-resistant and corrosion-resistant alloy materials [3], thereby enhancing the surface properties of the steel. Traditional cladding alloy materials are mainly based on nickel, with the addition of high-melting-point and high-hardness elements like W [4] and Ti [5], as well as ceramic-reinforced particle phases such as TiC [6,7], SiC [8], WC [9], Al_2_O_3_ [10,11], and TiO_2_ [12]. Yeh [13] introduced the concept of high-entropy alloys (HEAs), typically refers to new alloys containing five or more primary elements, with a molar fraction between 5% and 35%. HEA is a novel material, which is a combination of multiple principal elements in high concentrations. Compared to conventional alloys, HEAs exhibit lattice distortion, high entropy, as well as sluggish diffusion and “cocktail” effects [14,15], resulting in corrosion resistance, thermal stability, superior hardness, wear resistance, and mechanical properties [16], thereby attracting significant researcher interest. Currently, surface cladding technologies mainly include plasma arc welding [17], thermal spraying [18], cold spraying [19], plasma spraying [20], magnetron sputtering [21], and laser cladding [22]. Compared to other surface cladding techniques, laser cladding technology offers high energy density, fast cooling rates, efficient performance, minimal dilution rates, and excellent alloying capabilities with the substrate in the cladding process. Consequently, it is increasingly favored by researchers. An innovative method for improving the surface hardness, wear resistance, as well as corrosion resistance of steel is achieved by depositing high-melting-point, high-hardness, and corrosion-resistant high-entropy alloys on steel surfaces using laser cladding technology [23,24,25,26]. Employing high-speed laser cladding, Chong et al. [27] coated the surface of AISI 1045 steel with an AlCoCrFeNi HEA to enhance its surface hardness, wear resistance, and corrosion resistance. The treatment resulted in a 100 HV_0.2_ increase in hardness, a 0.15 decrease in friction coefficient, and a 9.85% reduction in wear rate. To improve the wear resistance, Hao et al. [28] utilized laser cladding technology to coat the Ti-6A-4V substrate with an AlTiVCrNb HEA coating. The results showed that the microhardness of the HEA cladding coating reached 548.54 HV_0.1_, which is 1.57 times that of the Ti-6A-4V substrate, resulting in a 1.58-fold improvement in wear resistance, significantly enhancing the wear resistance of the substrate material. Similarly, Chen et al. [29] used laser cladding technology to coat the 45 steel substrate with a WMoTaNb HEA coating. The results indicated that the microhardness of the cladding coating is higher than that of the substrate material, reaching 551 HV_0.2_. At room temperature, its friction coefficient remains within the range of 0.55 to 0.6, and at a high temperature of 800 °C, the friction coefficient increased to 0.8–1.0. Zhou [30] and Liu et al. [31] proposed that the inclusion of specific elements in particular HEA systems could boost their overall characteristics, including wear and corrosion resistance, by increasing lattice distortion. An investigation was conducted by Liao et al. [32] to analyze how the presence of vitriol (V) affects the microstructure, wear resistance, and corrosion resistance of an AlCoCrMoV*_x_*(*x* = 0, 0.2, 0.4, 0.6, 0.8, 1.0) HEA coating applied to 904L stainless steel. The results revealed that microhardness increased with V content, reaching a peak of 942.06 HV_0.1_. Initially, there was an increase in the friction coefficient, followed by a subsequent decrease, with the lowest wear rate at *x* = 0.8 and improved corrosion resistance with V addition. Liu et al. [33] enhanced the friction properties of FeCoCrNi HEA coatings on 45 steel by adding different amounts of Al, showing that microhardness increased from 202 to 546 HV_0.2_, and wear rate showed a decline from 8.55 × 10^−7^ mm^3^/(Nm) to 8.55 × 10^−9^ mm^3^/(Nm), significantly improving wear resistance. Zuo et al. [34] prepared CoCrFeNiTi*_x_* (*x* = 0, 0.2, 0.4, 0.6, 0.8) HEA coatings on 45 steel using laser cladding, finding that the microstructure changed from dendritic to equiaxed grains with increased Ti content, and microhardness reached a maximum of 502.39 HV_0.3_ at *x* = 0.8, with significantly improved corrosion resistance. However, research on adding W to FeCoCrNiAl HEAs is still limited, and the mechanisms are unclear, necessitating systematic investigation.

In summary, to improve the corrosion resistance, surface hardness, and wear resistance of 45 steel and expand its application scope, this study designs FeCoCrNiAlW*_x_* (*x* = 0, 0.2, 0.4, 0.6, 0.8) HEAs. Using laser cladding technology, FeCoCrNiAlW*_x_* HEA coatings are applied to the surface of 45 steel. The investigation delves into the impact of W content on the microstructure and characteristics of the coatings to unveil the underlying mechanisms, providing support for further expanding the applications of 45 steel.

## 2. Experimental Materials and Methods

### 2.1. Experimental Materials

The alloy 45 steel was selected as the substrate for HEA powder cladding. A 10 mm thick plate of 45 steel was cut into dimensions of 100 mm × 100 mm. After surface grinding, the substrates are polished sequentially with 200#, 400#, 600#, 800#, and 1800# grit sandpapers, cleaned with anhydrous ethanol, and air-dried in the laboratory for later use. The 99.9%-pure W powder with a particle size of 250 mesh was mixed with FeCoCrNiAl HEA powder (99.9% pure, 250 mesh particle size) in varying doping molar ratios. The FeCoCrNiAl HEA was pre-manufactured before the addition of W and was purchased from Zhuyu New Material Technology Co., Ltd. (Yangzhou, China). The W was added to the FeCoCrNiAl HEA for mixing. The mixture was homogenized using a planetary ball mill. During the ball milling process, the wet milling method was used with the addition of 50 mL of anhydrous ethanol to the grinding tank. Zirconia balls are used as grinding balls with a balls-to-powder ratio of 10:1. The rotation speed is 200 rpm, and the ball milling time is 3 h. The ball milling was conducted under normal room temperature conditions without the use of protective gas. Table 1 presents the compositions of the HEA powders at different element molar ratios.

### 2.2. Experimental Methods

**Figure 1:**
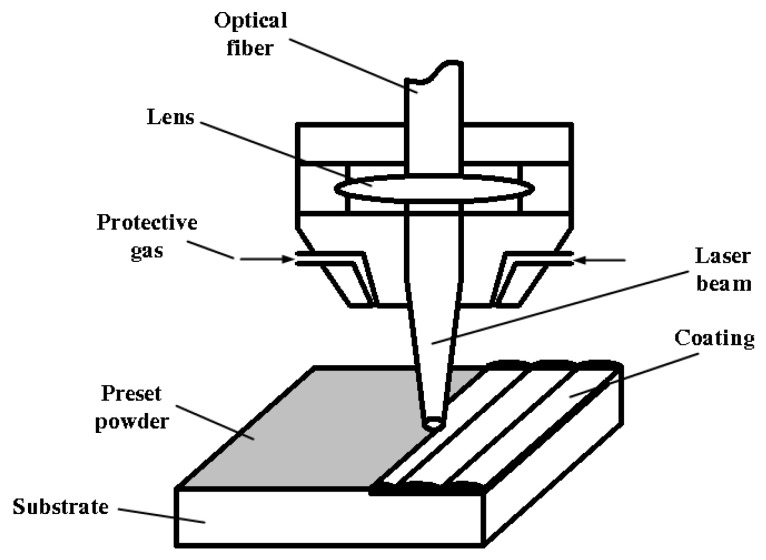
Schematic diagram of laser cladding with preset powder.

**Figure 2:**
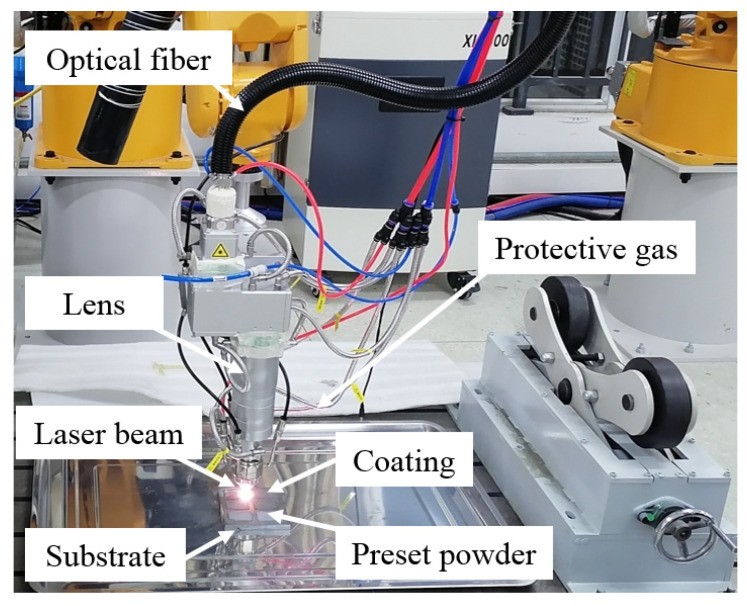
Laser cladding system.

Figure 1 illustrates the basic principle of laser cladding by preset powder in the experiment. The substrate was pre-coated with HEA powder, and a high-energy laser beam was moved at a specific velocity to melt the HEA powder onto the substrate under the protection of argon inert gas. To facilitate uniform pre-placement of the HEA powder on the substrate, a 2 mm thick acrylic template was designed. First, the HEA powder was stirred into a paste using a 5% polyvinyl alcohol solution and then pre-placed on the 45 steel substrate surface through the acrylic template to a thickness of 2 mm. Then, the substrates were dried at 70 °C for 24 h before laser cladding. The optimal laser power, laser spot diameter, scanning speed, overlap rate, and argon gas flow rate were determined by the overall macroscopic quality of the cladding coating. The experiment was performed using the Lantu laser cladding system (Haitian Laser Technology Co., Ltd., Ningbo, China). The process parameter values are detailed in Table 2. Both single-track and multi-track claddings were performed on the pre-placed coatings, as shown in Figure 2, which shows the actual laser cladding system and cladding process. This laser cladding system employs a coaxial powder feeding system, which offers several advantages over the off-axis method: better inert gas protection of the melt pool, a smoother cladding surface, simplified post-processing procedures, and reduced machining requirements. The CNC wire-cut electrical discharge machine (Model of BM400, Suzhou Baoma Numerical Control Machine Co., Ltd., Suzhou, China) was used to cut the single and multiple clad samples after cladding into different-sized small specimens, which were then used for various characterization and performance tests. The specimens were prepared for metallographic examination, polished sequentially with 200#, 400#, 800#, 1000#, and 1800# sandpapers, followed by polishing with 0.5-micron diamond suspension, and etched with 4.0% hydrofluoric acid solution for microstructure observation. The phase structure analysis of the cladding coating utilized a Bruker X-ray diffractometer from Germany( Bruker company, Karlsruhe city, Germany), with the diffraction speed set at 10°/min and a diffraction range of 20°–90°. A SEM3200 scanning electron microscope was used to observe the microstructure of the cladding coating, which is produced by China’s Guoyi Quantum Company (Hefei, China), and an Oxford Explorer 30 energy-dispersive spectrometer (Oxford Instruments, Oxford, UK) was used to analyze the composition. The HXS-1000AY Vickers hardness tester (Shanghai Haowei Optoelectronics Technology Co., Ltd., Shanghai, China) was used to measure the microhardness of the cladding coating, with a load mass of 0.2 kg and a loading time of 10 s, along the surface of the cladding coating to the substrate. Measurements of microhardness were taken at 0.1 mm intervals, with four parallel sets of data for each sample. The average value was taken as the hardness value of the sample. The wear resistance of the cladding coating was tested using a GF-I reciprocating wear and friction testing machine (Lanzhou Zhongke Kaihua Technology Development Co., Ltd., Lanzhou, China). The counterpart of the friction and wear test was a 5 mm diameter Si_3_N_4_ ceramic ball (Jinzhan bearing Co., Ltd., Shanghai, China); the test load was set to 5 N, the sliding distance was 5 mm, the sliding linear speed was 100 mm/s, and the test duration was 20 min. An electrochemical workstation (Shanghai Chenhua Instrument Technology Co., Ltd., Shanghai, China) was utilized for electrochemical corrosion experiments. Silicone insulation was applied to the non-clad surface of the sample, leaving only a 1 cm^2^ area of the HEA cladding coating exposed. The prepared specimens were submerged in a 3.5 wt.% NaCl solution for a duration of one hour then connected to the working electrode of the electrochemical workstation with a copper wire. Once the open circuit potential had reached a stable state, the scanning rate was configured at 5.0 × 10^−^⁴ V/s, and the sampling interval was set to 1.0 × 10^−^⁴ V and a resting time of 10 s for the electrochemical tests.

## 3. Results and Analysis

### 3.1. Macroscopic Morphology Analysis of Cladding Coating

**Figure 3:**
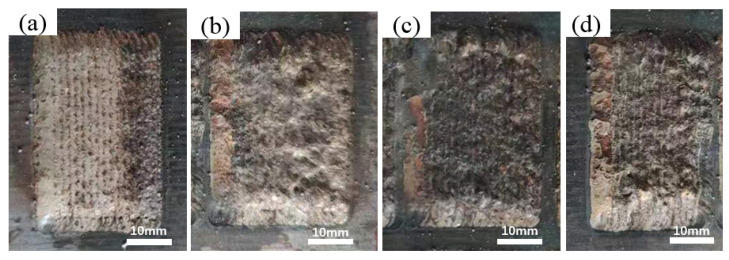

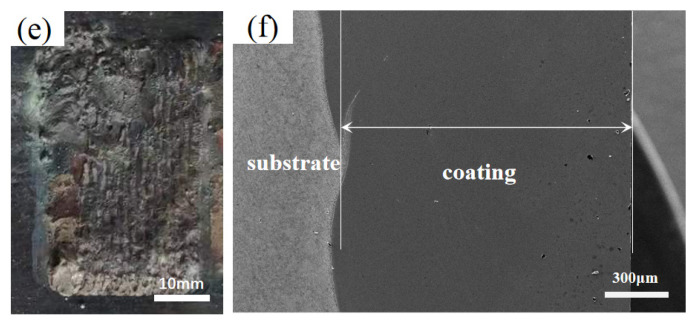
Surface macrophology and thickness of FeCoCrNiAlW*_x_* HEA cladding coatings. (**a**) *x* = 0; (**b**) *x* = 0.2; (**c**) *x* = 0.4; (**d**) *x* = 0.6; (**e**) *x* = 0.8; (**f**) cross section.

Figure 3 shows the macroscopic surface morphology of FeCoCrNiAlW*_x_* HEA multi-track laser-deposited coatings. From the figure, the surface morphology of the HEA coatings without added W demonstrates an overall good formation quality. However, as W content increases, the macroscopic surface morphology quality of the HEA coatings gradually deteriorates. The main reason is that the increase in W content leads to a reduction in wettability between the cladding coating and the substrate, as well as between different layers of the coating. On the other hand, with the increase in W content, the surface tension increases and a rough and uneven surface is formed, which further affects the forming precision. Additionally, it can be noted that there is a phenomenon of darkening on the cladding coating surface. This is primarily due to the organic compounds present in the added polyvinyl alcohol binder, which undergo combustion during the laser sintering process and causes the surface to darken. Polyvinyl alcohol is a common synthetic resin, whose main component is vinyl alcohol monomer. It is made of carbon–carbon bonds and carbon–hydrogen bonds, which are converted into carbon dioxide and water vapor during laser cladding. Due to the relatively fast cladding speed, the incompletely burned carbon particles of PVA in the cladding layer attach to the surface of the cladding coating, causing the surface to turn black. Figure 3f shows the cross section between the substrate and the coating. After the surface of coating has been ground down, the thickness of the cladding coating is approximately 1.54 mm.

### 3.2. Phase Analysis

**Figure 4:**
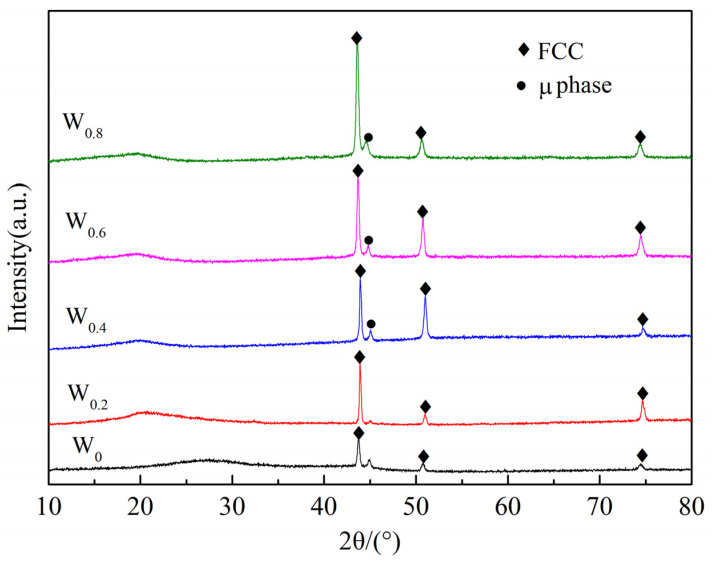
X-ray diffraction pattern of FeCoCrNiAlW*_x_* HEA cladding coating.

Figure 4 presents the X-ray diffraction (XRD) patterns of laser-deposited coating of FeCoCrNiAlW*_x_* (*x* = 0, 0.2, 0.4, 0.6, 0.8) HEAs. When W is not added, the FeCoCrNiAl cladding coating consists of a single face-centered cubic (FCC) solid-solution phase. FCC solid-solution alloys can be formed from the elements of Ni, Co, Fe, Mn, and Cr in different composition combinations [35]. After adding W to the HEA, the composition of the cladding coating gradually transitions from a single FCC solid solution to an FCC + µ phase (Fe_7_W_6_) with the increase in the W content, consistent with the conclusions of ref. [36]. During the laser cladding process, due to the rapid heating and cooling rates, the solute diffusion rate is much lower than the solidification rate, making it difficult to achieve equilibrium solidification. The fast cooling rate hinders the diffusion of W atoms, causing some W atoms to enter the solid-solution lattice and form a substitutional solid solution. Additionally, another portion of W atoms combines with Fe atoms to form a new compound precipitate (Fe_7_W_6_). The intermetallic phase Fe_7_W_6_ was called μ phase, which forms via a peritectic reaction of W with Fe [37]. The Fe_7_W_6_ phase contained an average W concentration of ~27 ± 4% [38]. The diffraction peaks of the FCC phase remain relatively stable, but the peak intensity gradually increases. It can be inferred that higher W content enhances the formation of the FCC phase within the cladding coating. The alloying of W has been evidenced to be able to remarkably improve the mechanical properties of FCC when W is introduced; multiple strengthening mechanisms, including solid-solution strengthening, precipitation strengthening (µ phase), and grain-refinement strengthening, have been discovered to be activated or enhanced. Nevertheless, some key scientific issues related to W doping in the FCC HEAs remain unknown. For example, quantitative descriptions of local lattice distortion, chemical composition fluctuation, and even short-range ordering caused by W doping are still lacking. For different precipitates, the thermodynamic and the kinetic characteristics of precipitation, as well as the processing parameters for accurately controlling the shape, size, and distribution of precipitate, are still unclear [39].

### 3.3. Microstructure Analysis

**Figure 5:**
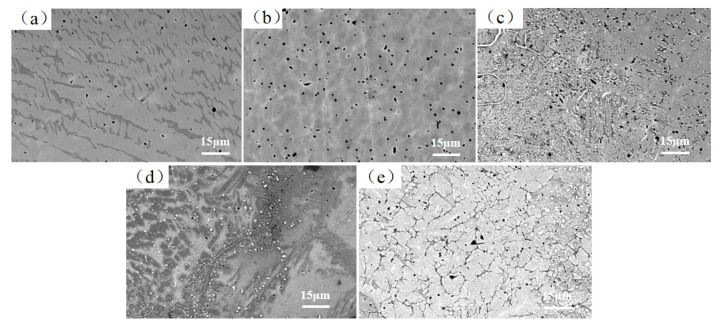
Microstructure and morphology of FeCoCrNiAlW*_x_* HEA cladding coating (1000×). (**a**) *x* = 0; (**b**) *x* = 0.2; (**c**) *x* = 0.4; (**d**) *x* = 0.6; (**e**) *x* = 0.8.

Figure 5 displays the microstructure morphology of the laser-deposited coating of the FeCoCrNiAlW*_x_* HEA. As seen in the figure, the predominant microstructure of the cladding coating comprises dendritic and cellular crystals. When *x* = 0, the microstructure is predominantly dendritic; when *x* = 0.6, the dendrites contain cellular crystals; and when *x* = 0.8, it is mainly composed of cellular crystals. This indicates that with the increase in W content, the microstructure gradually transitions from dendritic to cellular crystals. There are two primary reasons for this transition: first, the concentrated energy of laser cladding, rapid cooling rate, and high degree of supercooling enable rapid crystallization, inhibiting the anisotropic growth of crystals; second, W has a significantly higher melting point (3410 °C) compared to other elements in the FeCoCrNiAlW*_x_* HEA system. As the W content increases, the alloy system’s heat absorption capacity is further enhanced, which increases the solidification rate of the HEA, leading to grain refinement. At the same time, there are some porosities in the cladding coating. We think the main reason for this is taking polyvinyl alcohol as a binder, which was not fully removed during the drying process. During laser cladding, polyvinyl alcohol burns and decomposes into carbon monoxide, carbon dioxide, and water vapor. The rapid cooling rate of laser cladding prevents these gases from escaping, resulting in porosity. In future experiments, we plan to improve the experimental process by using methods such as vacuum degassing to reduce the porosity in the cladding coating.

**Figure 6:**
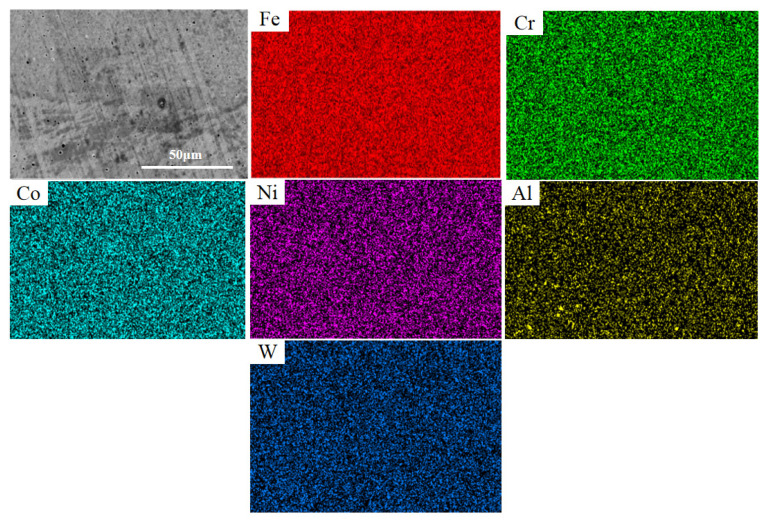
EDS mapping images of the FeCoCrNiAlW_0.4_ HEA cladding coating (500×).

Figure 6 shows the energy-dispersive spectroscopy (EDS) map of the FeCoCrNiAlW HEA cladding coating when *x* = 0.4. The alloying elements are distributed relatively uniformly throughout the cladding coating, and the added W is also uniformly distributed throughout the cladding coating. This indicates the feasibility of incorporating W into the FeCoCrNiAl HEA system, ensuring a uniform distribution of the alloying elements in the cladding coating, thereby preserving the overall uniformity in the performance of the cladding coating.

### 3.4. Microhardness Analysis

**Figure 7:**
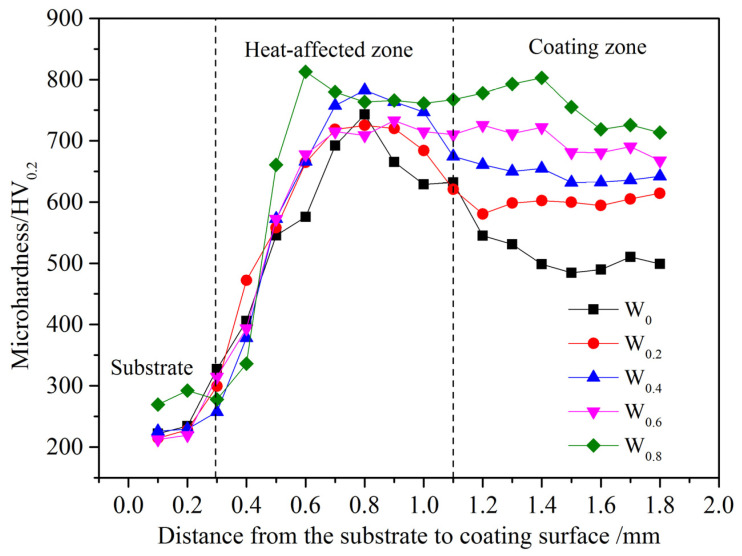
Microhardness of FeCoCrNiAlW*_x_* HEA cladding coating.

As shown in Figure 7, the cladding coating transitions into the substrate, heat-affected zone, and cladding zone from the base material to the surface. The figure shows that as the W content increases, the microhardness of the FeCoCrNiAlW*_x_* HEA cladding coating exhibits a gradual increase. Both the heat-affected zone and the cladding zone exhibit significantly higher microhardness than the substrate, with the former slightly surpassing the latter in microhardness. As the W content increases from *x* = 0 to *x* = 0.8, the average hardness of the cladding zone rises to 756.83 HV_0.2_ from 523.85 HV_0.2_. Compared to the substrate’s average hardness of 254.89 HV_0.2_, microhardness increased by 2.06 and 2.97 times in the cladding zone. The microhardness peaks at 812.77 HV_0.2_ in the heat-affected zone. Table 3 shows the average microhardness values of different HEA cladding coatings. The average microhardness of HEA cladding coatings prepared in this experiment are mostly higher than that of other HEA cladding coatings. The significant increase in microhardness can be attributed to several factors:

(1) The atomic radii of these elements are 124.6 pm (Ni), 124.1 pm (Fe), 124.9 pm (Cr), 125.1 pm (Co), 143 pm (Al), and 137 pm (W). The atomic radius of W is relatively large. When it enters the lattice to form substitutional solid solutions, it induces lattice distortion. As the W content increases, the lattice distortion effect gradually intensifies, leading to significant solid-solution strengthening and thereby increasing the hardness of the cladding layer.
(2) The laser cladding process, with its high energy density and rapid cooling rate, leads to a pronounced grain refinement effect during the alloy solidification process.
(3) The inclusion of W encourages the development of µ phase, increases dislocation slip resistance, and to some extent inhibits grain growth [40]. This leads to further grain refinement, resulting in an increase in the microhardness of the cladding coating.

**Table 3:** Microhardness of different HEA cladding coatings.

HEAs	Average Microhardness	Ref.
FeCoCrNiAlW_0.0_	523.85/HV_0.2_	This work
FeCoCrNiAlW_0.2_	601.99/HV_0.2_	This work
FeCoCrNiAlW_0.4_	647.90/HV_0.2_	This work
FeCoCrNiAlW_0.6_	698.82/HV_0.2_	This work
FeCoCrNiAlW_0.8_	756.83/HV_0.2_	This work
CoCrFeNiW_0.8_	432.02/HV_0.3_	[35]
CoCrFeNiW_0.75_	303.6/HV_0.2_	[41]
Al_1.0_FeMnNiCrCu_0.5_	541/HV_0.2_	[42]
FeMnNiCrCu_0.5_	193/HV_0.2_	[42]
CoCrFeNiTi_0.8_	502.39/HV_0.3_	[34]
CoCrFeNiSi_2.0_	566.5/HV_0.5_	[43]
FeCoNiCrSiAl_1.0_	439/HV_0.2_	[44]
AlCrFeCoNi	421/HV_0.2_	[45]
CoCrFeNiW	378/HV_0.2_	[46]

### 3.5. Friction and Wear Properties

**Figure 8:**
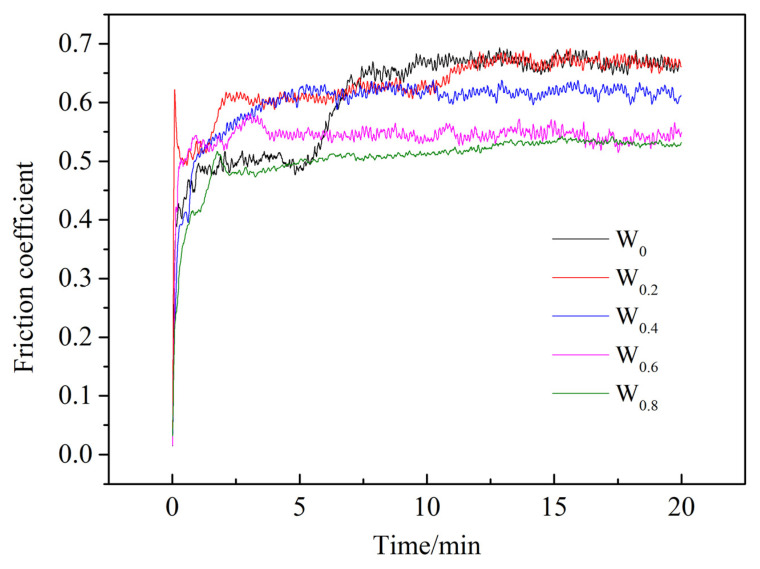
Friction coefficient curve of FeCoCrNiAlW*_x_* HEA cladding coating.

Figure 8 illustrates the FeCoCrNiAlW*_x_* high-entropy alloy cladding coating with friction coefficient curves, as the W content increases from *x* = 0 to *x* = 0.8. The friction coefficients of the cladding coatings with different W compositions display significant fluctuations during the running-in stage of the friction experiment, gradually stabilizing over time. During the stable phase, as the W content rises from *x* = 0 to *x* = 0.8, the average friction coefficients of the cladding coatings are 0.669, 0.664, 0.617, 0.544, and 0.527, respectively. Overall, the friction coefficient decreases with increasing W content, indicating that the incorporation of W improves the wear resistance of the cladding coating. It is noteworthy that the friction coefficients for *x* = 0 are similar with *x* = 0.2, suggesting minimal enhancement of wear resistance with low W content. However, the friction coefficients for *x* = 0.6 are close with *x* = 0.8; this suggests that a further increase in W content results in a saturation effect on enhancing wear resistance.

**Figure 9:**
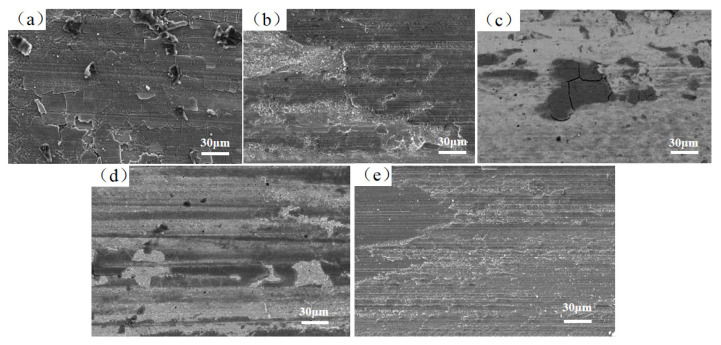
Worn morphologies of FeCoCrNiAlW*_x_* HEA cladding coating. (**a**) *x* = 0; (**b**) *x* = 0.2; (**c**) *x* = 0.4; (**d**) *x* = 0.6; (**e**) *x* = 0.8.

Figure 9 shows the wear morphology of the laser-deposited coatings of FeCoCrNiAlW*_x_* HEAs. At *x* = 0, the cladding coating surface exhibits significant cracking and spalling, with large chunks of the cladding coating detaching. This is primarily attributed to the comparatively lower hardness of the cladding coating. Under the reciprocating sliding load from the Si_3_N_4_ ball counterpart, the cladding coating undergoes fatigue detachment and transfer, resulting in the formation of pits. As the W content rises from *x* = 0.2 to *x* = 0.8, cracking and delamination in the cladding coating diminish. Additionally, the size of pits significantly reduces, weakening the micro-cutting action of abrasive particles on the coating surface and leading to a reduction in the friction coefficient. The main wear mechanisms are adhesive wear and abrasive wear, coupled with a small amount of spalling debris. This is because the microstructure of the cladding coating transitions from dendritic to cellular crystals and includes partial µ phase with the increase in W content, leading to smaller grain sizes, increased grain boundaries, and numerous bonding interfaces between grains. Additionally, the incorporation of µ-phase hard precipitates not only significantly increases the hardness of the cladding coating but also enhances its wear resistance [47].

W can promote the formation of µ phase, and with the increase in W content, the µ phase in the coating is more and more. The µ phase is a hard phase, which can effectively inhibit dislocation movement, increase the resistance of dislocation slip, realize the second-phase strengthening, increase the hardness of the cladding layer, and then improve the wear resistance of the cladding layer [35].

### 3.6. Corrosion Resistance Analysis

**Figure 10:**
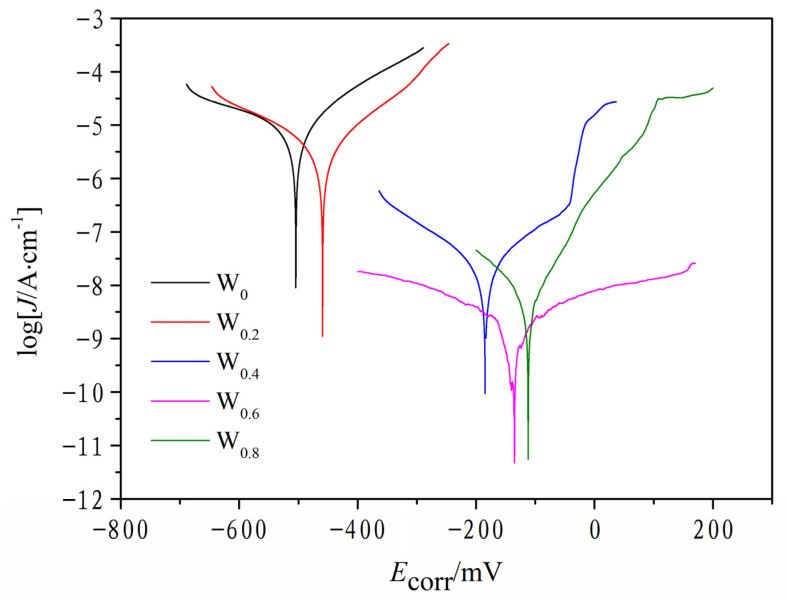
Potential polarization curve of FeCoCrNiAlW*_x_* HEA cladding coating.

Figure 10 illustrates the results that the electrochemical corrosion resistance experiments conducted on FeCoCrNiAlW*_x_* HEA laser-deposited coatings in the 3.5 wt.% NaCl solution with *x* ranging from 0 to 0.8. Through fitting, the corrosion potential and corrosion current density data for HEA coatings with different W contents were obtained, as shown in Table 4. The corrosion potential and current density reflect the corrosion characteristics of the HEA coating: a higher current density and lower corrosion potential indicate a stronger corrosion tendency and poorer corrosion resistance of the material; conversely, it indicates that the HEA coating has good corrosion resistance.

From Table 4, it is observed that as the W content increases, the corrosion potential of the FeCoCrNiAlW*_x_* HEA coatings rises, progressively, ranging from −515 mV to −112 mV. At the same time, the corresponding corrosion current density shows a general downward trend. Specifically, compared to the FeCoCrNiAlW*_x_* coatings with *x* = 0 and *x* = 0.2, the corrosion current density of the HEA coating with *x* = 0.4 is two orders of magnitude lower. Similarly, the HEA coatings with *x* = 0.6 and *x* = 0.8 exhibit corrosion current densities one order of magnitude lower than *x* = 0.4. Notably, the corrosion current density at *x* = 0.8 is similar to that at *x* = 0.6, indicating a saturation or diminishing return effect with increasing W content.

This indicates that the addition of W not only greatly enhances the corrosion resistance of the FeCoCrNiAl alloy system but also enhances the corrosion resistance of the FeCoCrNiAlW*_x_* HEA cladding coating as the content of W increases. It can be inferred that after reaching a certain W content threshold, further additions W may not significantly enhance the corrosion resistance of the FeCoCrNiAlW*_x_* HEA. This result is consistent with ref. [48]. In a corrosive test environment, with the increase in W*_x_* content (*x* = 0 to 0.8), FECoCrNiAlW*_x_* HEA cladding coatings can more easily be passivated; further, their passivation films can more easily be formed after the anodic activation stage [49]. The passive film of HEA cladding coating with high W content has relatively higher quantities of the metallic state elements (Fe, Cr, and Mn) than that of HEA cladding coating with low W content, which improves the corrosion resistance of the cladding coating [48]. Additionally, Table 4 shows that the corrosion resistance of the HEA cladding coatings prepared in this experiment is higher than that of other HEA coatings, indicating a wider application potential.

The FeCoCrNiAlW*_x_* HEA coatings possess high hardness, good wear resistance, and excellent corrosion resistance. These properties make them suitable for applications in steel rolling (e.g., steel rollers), petroleum equipment (e.g., drill pipe heads), die making, and agricultural machinery (e.g., rotary tiller blades). Utilizing these coatings in such applications can enhance product performance and extend service life.

The working conditions of components are complex, especially in high-temperature environments, where higher demands are placed on the strength, hardness, wear resistance, and corrosion resistance of the components. Therefore, future research should focus on investigating the performance of HEAs under different high-temperature conditions to further expand their applications in high-temperature and other specialized environments.

**Table 4:** Electrochemical parameters of FeCoCrNiAlW*_x_* and other different HEA coatings.

HEAs	E_cor*r*_/mV	J_corr_/A·cm^−2^	Ref.
FeCoCrNiAlW_0.0_	−515	5.43 × 10^−6^	This work
FeCoCrNiAlW_0.2_	−456	3.46 × 10^−6^	This work
FeCoCrNiAlW_0.4_	−186	2.73 × 10^−8^	This work
FeCoCrNiAlW_0.6_	−134	1.62 × 10^−9^	This work
FeCoCrNiAlW_0.8_	−112	5.26 × 10^−9^	This work
CoCrFeNiTi_0.6_	−509	4.22 × 10^−5^	[34]
CoCrFeNiSi_2.0_	−598.6	6.06 × 10^−7^	[43]
FeCoNiCrMnW_1.0_	−282	2.74 × 10^−5^	[48]
CoCrFeNiAl_0.3_	−451	1.03 × 10^−5^	[50]

## 4. Conclusions

In this study, FeCoCrNiAlW*_x_*(*x* = 0.0, 0.2, 0.4, 0.6, 0.8) HEA cladding coatings were prepared using the laser cladding pre-set powder process. The following conclusions are drawn based on the analysis of its macroscopic morphology, microscopic morphology, structure, and properties:

(1) The FeCoCrNiAlW*_x_* HEA cladding coatings mainly consist of FCC solid solution and µ phase. The microstructure is primarily composed of dendritic and cellular crystals, with the transition from dendritic to cellular crystals occurring gradually as the W content increases, and its doped W is evenly distributed throughout the cladding coating.
(2) The microhardness of the FeCoCrNiAlW*_x_* HEA cladding coating increases with the increase in W content. When *x* = 0.8, the hardness reaches 756.83 HV_0.2_. The wear resistance of the cladding coating also improves accordingly, with the friction coefficient gradually decreasing. When *x* = 0.6 and *x* = 0.8, the friction coefficients are relatively close, with the average friction coefficient reaching a minimum of 0.527. The wear of the cladding coating mainly manifests as adhesive wear and abrasive wear.
(3) The increase in the W element content made a significant impact on the corrosion resistance of the FeCoCrNiAlW*_x_* HEA cladding coating in a 3.5 wt.% NaCl solution, showing an upward trend in corrosion potential while the current density decreased accordingly. This change directly indicates that the corrosion resistance of the HEA coating is significantly improved with the increase in W content.

## Figures and Tables

**Table 1:** Element molar ratio of FeCoCrNiAlW*_x_* HEAs (at.%).

HEAs	Fe	Co	Cr	Ni	Al	W
FeCoCrNiAlW_0.0_	20.00	20.00	20.00	20.00	20.00	0.00
FeCoCrNiAlW_0.2_	19.23	19.23	19.23	19.23	19.23	3.85
FeCoCrNiAlW_0.4_	18.52	18.52	18.52	18.52	18.52	7.4
FeCoCrNiAlW_0.6_	17.86	17.86	17.86	17.86	17.86	10.7
FeCoCrNiAlW_0.8_	17.24	17.24	17.24	17.24	17.24	13.8

**Table 2:** Process parameter values for laser cladding.

Parameter	Value
Laser power	1200 W
Scanning speed	5 mm/s
Overlap ratio	50%
Laser spot diameter	3 mm
Argon flow rate	12 L/min

## Data Availability

The original contributions presented in the study are included in the article, further inquiries can be directed to the corresponding author.
